# A Haemophilic Dengue Patient with Pleural Effusion and Earache

**DOI:** 10.7759/cureus.9572

**Published:** 2020-08-05

**Authors:** Rajesh Das, Md Prova Zaman Emon, Sharmin Akter Shanu, Dilruba Akter, Md Rabiul Islam

**Affiliations:** 1 Pharmacy, University of Asia Pacific, Dhaka, BGD; 2 Medicine, Sheikh Russel National Gastroliver Institute and Hospital, Dhaka, BGD

**Keywords:** dengue fever, pleural effusion, plasma leakage, haemophilia, earache, bangladesh

## Abstract

About 2.5 billion people are living at a higher risk of dengue fever in hundreds of tropical and sub-tropical countries. Treatment of dengue fever is quite complicated and challenging because of the lack of effective treatment approaches. We herein report a rare case of a 25-year-old female with a past medical history of haemophilia A, suffering from dengue fever, pleural effusion, earache, myalgia, headache, and vomiting. Dengue was confirmed by the non-structural protein 1 (NS1) antigen and immunoglobulin M (IgM) antibody test. She had low blood pressure (80/60 mmHg), frequent vomiting, and low platelet count during hospitalization. Moreover, a genetic disorder like haemophilia with plasma leakage and earache made the patient's condition worse. However, by repeated platelet infusion, the platelet counts elevated and the patient was discharged from the hospital after nine days. Complete recovery was achieved after 27 days. This is a rare case of dengue; physicians should be aware of the severity of the disease and its management tactics. More discussion and research need to be carried out to develop an effective and optimized treatment and management options to reduce the mortality and morbidity due to dengue fever with a co-morbid disease.

## Introduction

Dengue fever (DF) is a vector-borne, febrile, viral illness caused by the four dengue virus (DENV) serotypes of the Flaviviridae family, genus Flavivirus, namely, DENV-1, DENV-2, DENV-3, and DENV-4, the symptoms of which appear after three to 14 days of the first bite. DF is transmitted by arthropod mosquitoes, Aedes aegypti and Aedes albopictus [1]. Dengue is endemic in hundreds of different tropical and sub-tropical countries, where about 2.5 billion people are living at high risk. About 50-100 million people suffer from DF every year with approximately 22,000 deaths per year. The prevalence of DF has increased about 30 folds in the last 50 years and 40% of people are at risk of DF [2-3]. One of the effective diagnosis procedures for DF includes the non-structural protein 1 (NS1) antigen test with a sensitivity of 48.5%-58.6% and specificity of 92.5%-99.4%. The combined sensitivity of the NS1 and immunoglobulin M (IgM) antibody tests can increase up to 89.9%-92.9%, with a specificity of 75.0%-88.8% [4-5].

DF is a self-limiting disease with varieties of symptoms, including fever, myalgia, rash, leukopenia, and thrombocytopenia (a condition with low blood platelet count) [6]. Patients with pre-existing coagulopathy like haemophilia increase the risk of bleeding in a dengue patient because it prevents the development of a higher platelet count [7]. Haemophilia A is a genetic bleeding disorder caused by defects in the coagulation factor VIII gene and is an extremely rare disease (approximately 1:5000). It can be detected if the blood contains less clotting factor VIII [8]. Another complication of dengue fever arises from plasma leakage and pleural effusion. According to the World Health Organization (WHO), the diagnosis of plasma leakage is difficult and can be measured by serious pleural effusion. Patients with pleural effusion should be treated with care because plasma leakage can develop cardiorespiratory dysfunction in 5.7% of patients with a mortality rate of 7.3% [9-10]. We herein present a haemophilic patient suffering from dengue fever, pleural effusion, and earache managed at a hospital in Dhaka, Bangladesh.

## Case presentation

A 25-year-old woman presented with four days of moderate to high fever (104^o^C) and headache followed by progressive weakness. On the third day, she was suffering from vomiting five times in three hours, which ultimately weaken the patient. After four days of fever and headache, she consulted a doctor who had prescribed her paracetamol and advised to perform some clinical investigations such as complete blood count (CBC), dengue NS1, and the Widal test. The Widal test report was found to be negative, but the dengue NS1 test was reported positive. After consultation with the doctor, she got admitted to the hospital.

**Figure 1:**
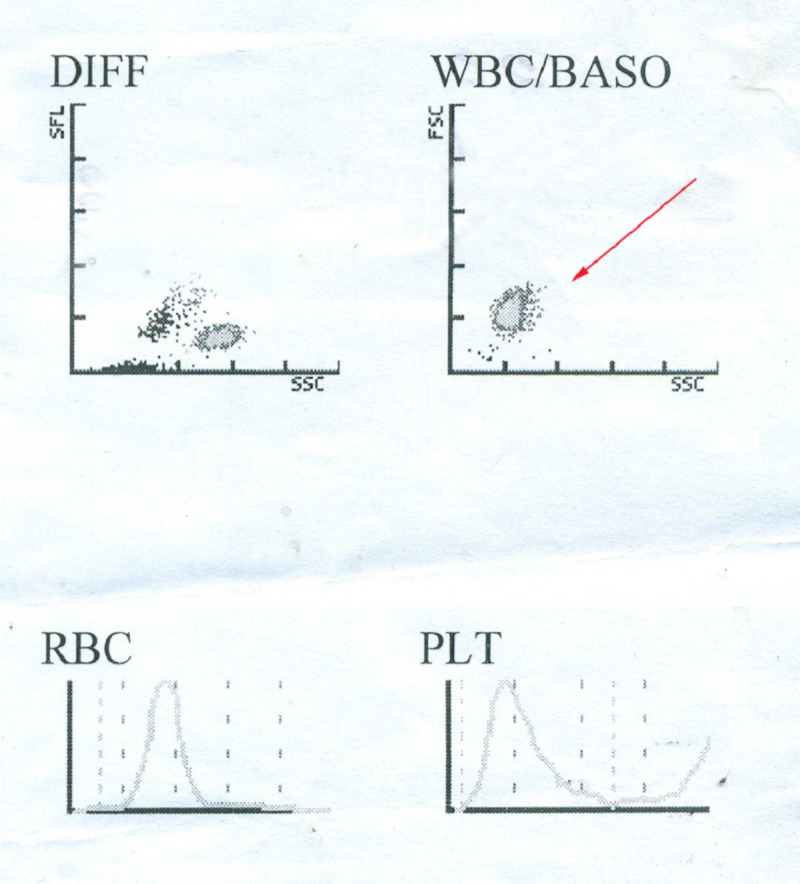
Automatic cell count during hospitalization on day two Lymphopenia and leukocytopenia marked by the red arrow (top-right) BASO: Basophil; DIFF: Differential; FSC: Forward scatter; PLT: Platelet; RBC: Red blood cell; SFL: Side fluorescence; SSC: Side scatter; WBC: White blood cell

After hospitalization, all the tests were conducted again, including the immunoglobulin M (IgM) antibody test and the factor VIII test. The results confirmed DF along with haemophilia A. On examination, the patient was found to be severely dehydrated with low blood pressure (60/85 mmHg) and was managed with continuous intravenous infusion of a multivitamin containing normal saline. Paracetamol was continued as the only drug for the treatment but headache and body pain were not alleviated. Platelet count was decreasing rapidly and due to continuous intravenous (IV) infusion, the patient developed cold and nasal congestion. Xylometazoline hydrochloride 0.1% drop was prescribed to treat nasal congestion, as the patient experienced mild breathing problems. On the second day, after the automatic cell counting test, the patient was found to be suffering from lymphopenia and leukocytopenia (Figure 1).

The different haematological parameters were monitored continuously during the hospital stay (Table 1).

**Table 1:** Results of different haematological parameters of the case on different days after hospitalization D: Day of hospitalization; E: Evening; ESR: Erythrocyte sedimentation rate (Westergren); Hb: Haemoglobin; L: Lymphocytes; M: Morning; N: Neutrophils; PCV: Packed cell volume; WBC: White blood cell

Parameters	D1	D2	D3	D4 M	D4 E	D5 M	D5 E	D6	D7	D8	D9	D11
Platelets (x10^Ù^^9^/L)	240	142	68	31	14	17	30	22	35	60	117	217
Hb (g/dl)	12.1	11.8	13.8	14.8	14.6	13.2	14.2	11.5	11.6	11.7	12.4	12.0
ESR (mm/h)	27	21	2	4	2	2	4	10	25	25	33	42
WBC (x10^Ù^^9^/L)	6.0	2.36	3.29	6.0	11.0	15.0	21.0	11.38	11.84	9.0	7.86	10.1
N (%)	92	82	62	58	50	62	51	73	83	74	57	59
L (%)	5	15	32	35	42	30	42	21	13	20	38	32
PCV (%)	37.3	36.8	42.7	45	43.8	40.2	43.6	35.6	35.0	36.4	39.0	36.2

**Figure 2:**
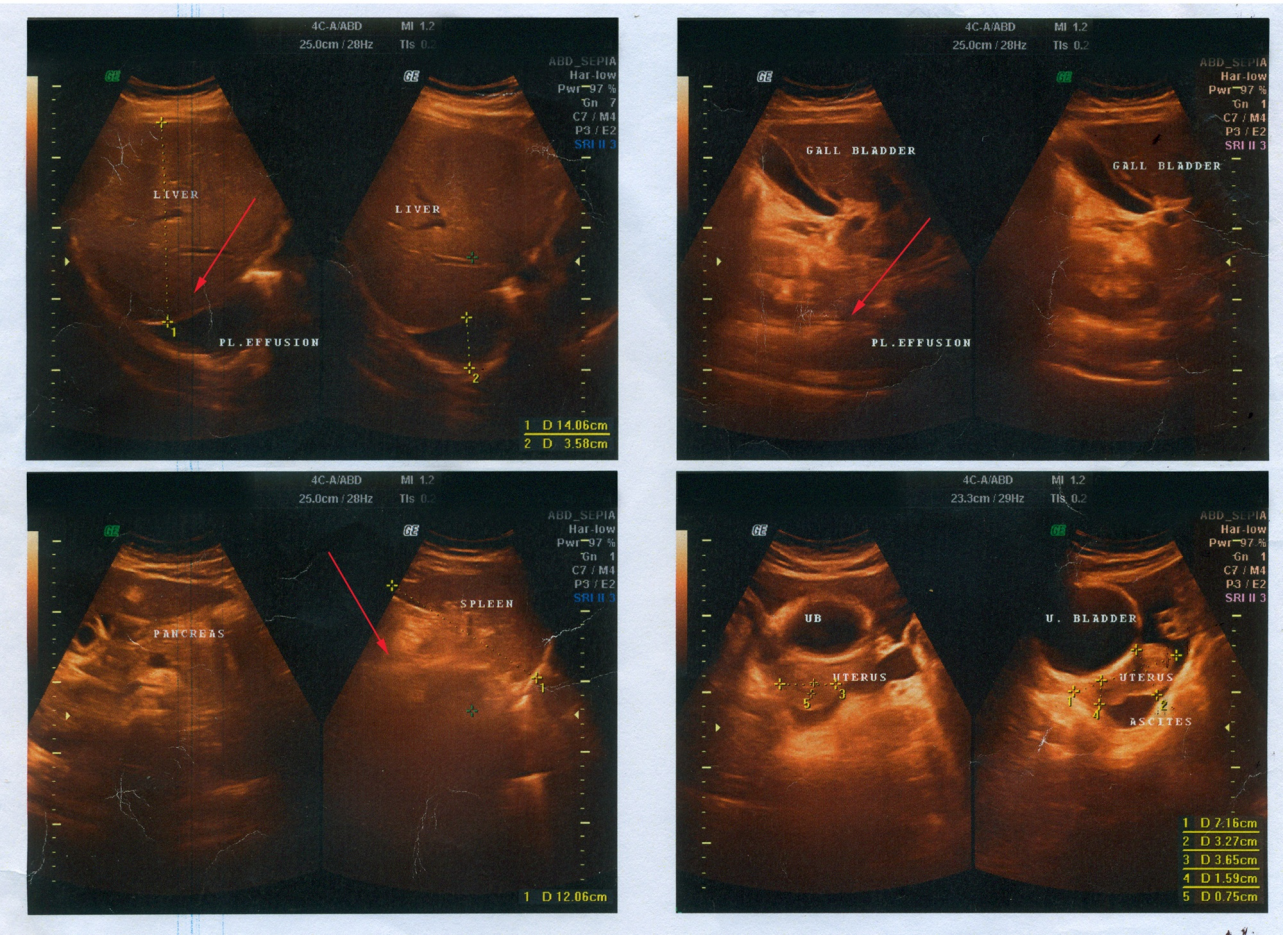
Ultrasonogram of the whole abdomen during hospitalization at day four Mild hepatomegaly with fatty infiltration of the liver (top-left), splenomegaly (bottom-left), pleural effusion and abdominal ascites (top-left and top-right) marked by red arrows.

On the fourth day, in the morning, the platelet level dropped to 31 x 10^9/L and the patient experienced bleeding from the nose with a mild earache. Tranexamic acid 100 mg/ml was administered to reduce the bleeding, which stopped completely after two days. An ultrasonogram of the whole abdomen revealed mild hepatomegaly with infiltration of the liver, splenomegaly, abdominal ascites, and right-sided pleural effusion (Figure 2).

The patient’s earache gradually intensified. CBC reports that were performed on the evening of the same day showed the platelet count to be 14 x 10^9/L. To manage the gradually deteriorating condition, platelets from blood donors were infused to the patient.

**Figure 3:**
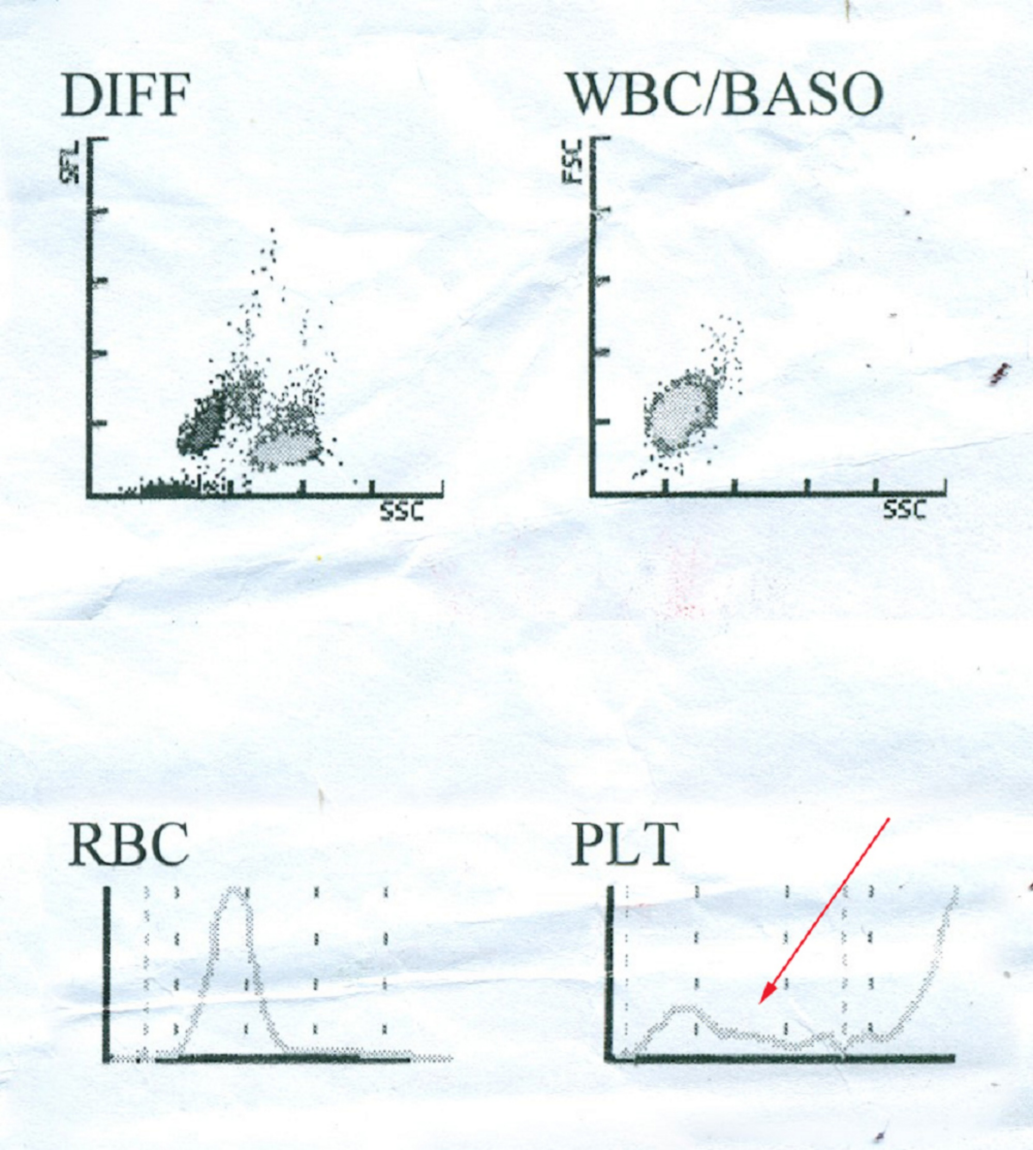
Automatic cell count during hospitalization at day six Abnormal platelet distribution and thrombocytopenia marked by the red arrow (bottom-right). BASO: Basophil; DIFF: Differential; FSC: Forward scatter; PLT: Platelet; RBC: Red blood cell; SFL: Side fluorescence; SSC: Side scatter; WBC: White blood cell.

On the fifth day, platelets started to increase but earache in the right ear of the patient did not improve. The doctor assumed that plasma leakage in the middle ear is responsible for the development of the earache. Antibiotics were not prescribed due to DF and low platelet count. On the sixth day, the platelet count again started to fall. Serum glutamic pyruvic transaminase (SGPT) was found to be above normal (82 U/L; reference range = up to 31 U/L). Again, platelets were infused to the patient. An automatic call counting test was then performed, which revealed no sign of lymphopenia and leukocytopenia but showed the development of thrombocytopenia with abnormal platelet distribution (Figure 3).

Finally, from the seventh day onward, the condition of the patient improved and platelet count elevated without any severe complications. On the ninth day of hospital stay, the patient was discharged and was advised complete bed rest of 15 days followed up by a CBC test. General medications were prescribed on discharge, including esomeprazole 20 mg twice daily for 15 days, domperidone 10 mg twice daily for 15 days, fexofenadine 120 mg once daily for seven days, paracetamol 665 mg thrice daily for three days, magaldrate plus simethicone combination suspension (10 ml after meal) for five days, miconazole (to be applied over the tongue after the meal; three times daily) for 15 days, and nystatin drop (15 drops to be applied over the tongue after the meal; three times daily) for 15 days. Antibiotic (cefuroxime + clavulanic acid 500/125 mg) was prescribed twice daily for 10 days for the treatment of earache. All the symptoms were finally resolved after completing the above medications.

## Discussion

In Bangladesh, from January to November 2019, 100,107 patients were admitted in the hospital and 129 died, which was worse than any past occurrence in the country (6,232 in 2002, 3,934 in 2004, 3,162 in 2015, 6,060 in 2016, and 10,148 in 2018) [11]. In the rainy season, water logging is very common in Bangladesh, which is a congruent environment for the propagation of mosquitos. Many of the affected patients died at that time due to inadequate knowledge of DF [12]. According to WHO guidelines, DF was classified as dengue without a warning sign, dengue with a warning sign, and severe dengue for the better management and treatment of dengue patients [13]. Many researchers suggested that vomiting for two or more consecutive days, or three times in one hour, or five times in six hours indicates persistent vomiting and severe dengue [14-15]. As our case vomited five times in three hours, her condition was severe.

As the patient was severely dehydrated due to persistent vomiting, intravenous (IV) saline with multivitamin was administered to manage dehydration, weakness, as well as improve blood pressure. Cold and nasal congestion were managed by xylometazoline nasal drop. According to Oliveira et al., leukopenia (68.3%), thrombocytopenia (66.5%), lymphocytopenia (67.2%), and atypical lymphocytes (67%) are very common in dengue patients, and these haematological abnormalities indicate the severity [16]. As platelet levels gradually dropped, the patient’s condition was deteriorating, which was managed by platelet infusion. The nose bleeding due to pleural effusion was managed with anti-fibrinolytic agents.

In the meantime, the patient experienced severe earache but any medication was not suggested except for paracetamol. Finally, the platelet count started to improve from the seventh day and after nine days of treatment, the patient was stable. Then oral antibiotic therapy (beta-lactam and beta-lactamase inhibitor) was prescribed for the management of her ear infection, and she was discharged from the hospital along with other necessary medication.

## Conclusions

This is a rare case and may be the first case of any hemophilic dengue patient having numerous complications like pleural effusion, earache, myalgia, headache, and vomiting. Because of such an escalating number of complicated cases that need to be managed with scarce resources, poor socioeconomic conditions, and inadequacy of public health awareness, the situation, recently, has become more challenging. Physicians should be very careful about treating dengue patients with haemophilia and pleural effusion as it is a life-threatening condition. Further discussion and research are necessary to determine the effective management of these kinds of cases to reduce and prevent the risk of dengue-related morbidity and mortality. Hospitals and physicians in the small towns of developing countries also need to be adequately equipped and trained for handling such complicated cases.
